# Supplementary material for: Allosteric regulation and crystallographic fragment screening of SARS-CoV-2 NSP15 endoribonuclease
Source: Nucleic Acids Res. 2023 Apr 28;51(10):5255–70. doi: 10.1093/nar/gkad314 (PMC10250223; doi:10.1093/nar/gkad314)
Supplement: gkad314_Supplemental_Files [file gkad314_supplemental_files.zip › Supplementary datasets.zip!/139/Refine_1/01-BUSTER/Cycle-1/shell.01/R_Rfree.html]

# Rxpct factors in resolution bins at cycle 0


|  |  |  |  |  |  |  |  |  |
| --- | --- | --- | --- | --- | --- | --- | --- | --- |
|  | **<d> (Å)**  Rxpct\_Work | Rxpct\_Free | Rxpct\_All | |  |  |  |  |
|  | 13.2733 | 0.3114 | 0.4374 | 0.3167 |  |
|  | 7.6634 | 0.2685 | 0.2706 | 0.2686 |  |
|  | 5.9360 | 0.2783 | 0.2573 | 0.2775 |  |
|  | 5.0168 | 0.2099 | 0.2007 | 0.2094 |  |
|  | 4.4244 | 0.1817 | 0.1693 | 0.1811 |  |
|  | 4.0021 | 0.1672 | 0.1490 | 0.1664 |  |
|  | 3.6814 | 0.1895 | 0.2114 | 0.1905 |  |
|  | 3.4272 | 0.1904 | 0.1725 | 0.1894 |  |
|  | 3.2193 | 0.1799 | 0.1826 | 0.1801 |  |
|  | 3.0451 | 0.1793 | 0.1779 | 0.1792 |  |
|  | 2.8965 | 0.1891 | 0.1848 | 0.1889 |  |
|  | 2.7677 | 0.1816 | 0.1837 | 0.1817 |  |
|  | 2.6547 | 0.1871 | 0.2023 | 0.1881 |  |
|  | 2.5545 | 0.1947 | 0.1880 | 0.1943 |  |
|  | 2.4648 | 0.1946 | 0.1930 | 0.1945 |  |
|  | 2.3840 | 0.2079 | 0.2242 | 0.2087 |  |
|  | 2.3106 | 0.2067 | 0.1873 | 0.2059 |  |
|  | 2.2436 | 0.2146 | 0.2015 | 0.2139 |  |
|  | 2.1821 | 0.2008 | 0.1911 | 0.2003 |  |
|  | 2.1254 | 0.2056 | 0.2012 | 0.2054 |  |
|  | **Overall** | 0.2041 | 0.2037 | 0.2041 |  |

# Rxpct factors in resolution bins at cycle 1


|  |  |  |  |  |  |  |  |  |
| --- | --- | --- | --- | --- | --- | --- | --- | --- |
|  | **<d> (Å)**  Rxpct\_Work | Rxpct\_Free | Rxpct\_All | |  |  |  |  |
|  | 13.2733 | 0.3113 | 0.4364 | 0.3166 |  |
|  | 7.6634 | 0.2677 | 0.2714 | 0.2680 |  |
|  | 5.9360 | 0.2771 | 0.2525 | 0.2762 |  |
|  | 5.0168 | 0.2088 | 0.2005 | 0.2083 |  |
|  | 4.4244 | 0.1813 | 0.1679 | 0.1806 |  |
|  | 4.0021 | 0.1669 | 0.1496 | 0.1661 |  |
|  | 3.6814 | 0.1894 | 0.2135 | 0.1906 |  |
|  | 3.4272 | 0.1892 | 0.1686 | 0.1881 |  |
|  | 3.2193 | 0.1791 | 0.1832 | 0.1794 |  |
|  | 3.0451 | 0.1778 | 0.1749 | 0.1776 |  |
|  | 2.8965 | 0.1874 | 0.1882 | 0.1874 |  |
|  | 2.7677 | 0.1809 | 0.1837 | 0.1811 |  |
|  | 2.6547 | 0.1864 | 0.2053 | 0.1875 |  |
|  | 2.5545 | 0.1934 | 0.1889 | 0.1931 |  |
|  | 2.4648 | 0.1927 | 0.1920 | 0.1927 |  |
|  | 2.3840 | 0.2076 | 0.2238 | 0.2084 |  |
|  | 2.3106 | 0.2062 | 0.1825 | 0.2051 |  |
|  | 2.2436 | 0.2140 | 0.2001 | 0.2133 |  |
|  | 2.1821 | 0.2010 | 0.1934 | 0.2007 |  |
|  | 2.1254 | 0.2070 | 0.2010 | 0.2067 |  |
|  | **Overall** | 0.2034 | 0.2033 | 0.2034 |  |

# Rxpct factors in resolution bins at cycle 2


|  |  |  |  |  |  |  |  |  |
| --- | --- | --- | --- | --- | --- | --- | --- | --- |
|  | **<d> (Å)**  Rxpct\_Work | Rxpct\_Free | Rxpct\_All | |  |  |  |  |
|  | 13.2733 | 0.3105 | 0.4333 | 0.3156 |  |
|  | 7.6634 | 0.2662 | 0.2707 | 0.2665 |  |
|  | 5.9360 | 0.2745 | 0.2538 | 0.2737 |  |
|  | 5.0168 | 0.2071 | 0.1999 | 0.2066 |  |
|  | 4.4244 | 0.1809 | 0.1695 | 0.1803 |  |
|  | 4.0021 | 0.1677 | 0.1490 | 0.1668 |  |
|  | 3.6814 | 0.1887 | 0.2120 | 0.1898 |  |
|  | 3.4272 | 0.1894 | 0.1672 | 0.1882 |  |
|  | 3.2193 | 0.1787 | 0.1860 | 0.1791 |  |
|  | 3.0451 | 0.1775 | 0.1762 | 0.1774 |  |
|  | 2.8965 | 0.1865 | 0.1850 | 0.1865 |  |
|  | 2.7677 | 0.1806 | 0.1814 | 0.1807 |  |
|  | 2.6547 | 0.1854 | 0.2014 | 0.1863 |  |
|  | 2.5545 | 0.1932 | 0.1887 | 0.1930 |  |
|  | 2.4648 | 0.1926 | 0.1865 | 0.1923 |  |
|  | 2.3840 | 0.2076 | 0.2255 | 0.2084 |  |
|  | 2.3106 | 0.2050 | 0.1828 | 0.2040 |  |
|  | 2.2436 | 0.2113 | 0.1999 | 0.2107 |  |
|  | 2.1821 | 0.2001 | 0.1976 | 0.2000 |  |
|  | 2.1254 | 0.2067 | 0.2046 | 0.2066 |  |
|  | **Overall** | 0.2027 | 0.2029 | 0.2027 |  |

# Rxpct factors in resolution bins at cycle 3


|  |  |  |  |  |  |  |  |  |
| --- | --- | --- | --- | --- | --- | --- | --- | --- |
|  | **<d> (Å)**  Rxpct\_Work | Rxpct\_Free | Rxpct\_All | |  |  |  |  |
|  | 13.2733 | 0.3011 | 0.4253 | 0.3063 |  |
|  | 7.6634 | 0.2508 | 0.2630 | 0.2516 |  |
|  | 5.9360 | 0.2553 | 0.2478 | 0.2550 |  |
|  | 5.0168 | 0.2020 | 0.2060 | 0.2023 |  |
|  | 4.4244 | 0.1759 | 0.1788 | 0.1760 |  |
|  | 4.0021 | 0.1691 | 0.1621 | 0.1688 |  |
|  | 3.6814 | 0.1848 | 0.2184 | 0.1864 |  |
|  | 3.4272 | 0.1849 | 0.1775 | 0.1845 |  |
|  | 3.2193 | 0.1749 | 0.1950 | 0.1760 |  |
|  | 3.0451 | 0.1726 | 0.1802 | 0.1730 |  |
|  | 2.8965 | 0.1818 | 0.1919 | 0.1823 |  |
|  | 2.7677 | 0.1768 | 0.1808 | 0.1770 |  |
|  | 2.6547 | 0.1832 | 0.2022 | 0.1843 |  |
|  | 2.5545 | 0.1885 | 0.1948 | 0.1888 |  |
|  | 2.4648 | 0.1883 | 0.1975 | 0.1888 |  |
|  | 2.3840 | 0.2010 | 0.2185 | 0.2018 |  |
|  | 2.3106 | 0.2000 | 0.1875 | 0.1995 |  |
|  | 2.2436 | 0.2089 | 0.2007 | 0.2085 |  |
|  | 2.1821 | 0.1975 | 0.1982 | 0.1976 |  |
|  | 2.1254 | 0.2067 | 0.1962 | 0.2062 |  |
|  | **Overall** | 0.1973 | 0.2072 | 0.1978 |  |
